# Bismuth (III) Salts Promoted and Ionic Liquid Assisted an Efficient and Environmentally Benign One-Pot Synthesis of 1,5-Benzodiazepine Derivatives

**DOI:** 10.5402/2011/604348

**Published:** 2011-05-14

**Authors:** Atul Chaskar, Latika Patil, Kiran Phatangare, Vikas Padalkar, Santosh Takale

**Affiliations:** ^1^Department of Chemistry, National Taiwan University, 106 Taipei, Taiwan; ^2^Department of Chemistry, C. K. Thakur Research Centre, Navi Mumbai 410206, India

## Abstract

1,5-Benzodiazepine derivatives were synthesized by the condensation reactions of *o*-phenylenediamine and ketones catalyzed by bismuth (III) salts under mild conditions. This method is easy, efficient, environment and eco-friendly, free of toxic catalysts, and gives good to excellent yields of 1, 5-benzodiazepines.

## 1. Introduction

The synthesis of 1,5-benzodiazepines and their derivatives have attracted considerable attention of researchers, including pharmaceutical and organic synthetic chemists, in recent years because of their medicinal properties namely antianxiety, hypnotic, antidepressive, tranquilizing, antiinflammatory, anticonvulsant, antifeedant, antibacterial, and analgesic agents [1–4]. In addition, 1,5-benzodiazepines are valuable synthons used for the preparation of other fused ring compounds such as triazolo, oxazino or furano-benzodiazepines [5, 6]. Benzodiazepines derivatives are also used in industry as dyes for acrylic fibers in photography [7].

Due to their wide range of pharmacological activities and industrial and synthetic applications, the development of practical and green protocols continues to be a challenging endeavour in synthetic chemistry. In recent years, many methods for their preparation are reported in the literature. These include condensation reactions of *o*-phenylenediamine with *α*, *β*-unsaturated carbonyl compounds [8], *β*-haloketones [9], *β*-aminoketones [10] or ketones promoted by BF_3_·OEt_2_ [11], NaBH_4_ [12], polyphosphoric acid or SiO_2_ [13], ceric ammonium nitrate (CAN) [14], MgO/POCl_3_ [15], Yb(OTf)_3 _ [16], Al_2_O_3_/P_2_O_5_ or AcOH under microwave conditions [17], Amberlyst-15 in ionic liquid [18], CeCl_3_/7H_2_O/NaI supported on silica gel [19], InBr_3_ [20], 1-butyl-3-methylimidazolium bromide ([bmim]Br) [21], Sc(OTf)_3 _ [22], and Nb(Cl)_3 _ [23]. However, many of these methodologies have one or more shortcomings, such as long reaction time, poor yields of the products, drastic reaction conditions, occurrence of several side products, expensive reagents, high catalyst loading, and tedious workup procedures.

Bismuth (III) salts have emerged in the recent years as “eco-friendly” reagents suitable for green chemistry. They have received considerable attention as mild Lewis acids [24–27] for an array of organic transformations because the catalysts are inexpensive, relatively nontoxic, moisture and air tolerant, environmentally benign, and commercially available. Ionic liquid is used as an alternative to traditional solvents for organic reactions particularly in the area of green chemistry. 

**Scheme 1:**
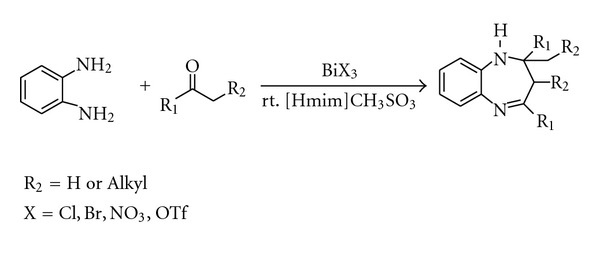


Thus, considering the advantages and applications of bismuth (III) salts and ionic liquid, and as part of our ongoing project to explore the catalytic activities of bismuth (III) salts in organic transformations [28], we attempted the convenient and practical synthesis of 1,5 benzodiazepines using bismuth (III) salts (Scheme 1).

In order to investigate the catalytic efficiency of bismuth (III) salts and compare it with different acid catalysts, the condensation of *o*-phenylendiamine with acetone in presence of different catalysts at room temperature was investigated, and results are shown in Table 1.

The results mentioned in Table 1 demonstrate the effective use of bismuth (III) salts and ionic liquid for the synthesis of 1,5-benzodiazepines.

Encouraged by the results obtained in the above reaction and in order to prove the generality and scope of this new protocol, a wide verity of ketones were evaluated and the results are summarised in Table 2. Aromatic, aliphatic, and cyclic ketones reacted with *o*-phenylenediamine, affording the corresponding 1,5-benzodiazepines in good to excellent yields. Cyclic ketones gave fused ring benzodiazepines. Products were characterized by ^1^H-NMR, ^13^C NMR, and Mass and physical constant. Physical and spectral data of known compounds are in good accordance with those reported in the literature.

Further, we extended this protocol to substituted *o*-phenylenediamine, and we have observed that the electron donating group increases the rate of reaction, whereas electron withdrawing group decreases the rate of reaction and yield of product.

## 2. Conclusion

In conclusion, we have demonstrated here a new and efficient procedure for the synthesis of 1,5-benzodiazepine derivatives catalyzed by bismuth (III) salts in ionic liquid. The advantages of our protocol are easy workup, fast reaction rate, and mild reaction condition with good yield, which make the method an attractive and a useful contribution to the present methodologies.

## 3. Experimental

All commercial reagents were used as received without purification, and all solvents were reagent grade. The reaction was monitored by TLC using on 0.25 mm E-Merck silica gel 60 F254 precoated plates, which were visualized with UV light. Melting points were taken in open capillaries. The IR spectra were recorded on a PerkinElmer 257 spectrometer using KBr discs. ^1^H NMR and ^13^C NMR spectra were recorded on a VXR-300 MHz instrument using TMS as an internal standard.

### 3.1. General Experimental Procedure

A mixture of *o*-phenylenediamine (0.01 mol), ketone (0.02 mol), and bismuth (III) salts (0.002 mol) in ionic liquid (2 mL) was stirred at room temperature for 1 hr. After completion of reaction, as monitored by TLC, the reaction mixture was extracted with ethyl acetate (2 × 10 mL). The combined organic extract were washed with distilled water, dried over Na_2_SO_4_, and removal of the solvent under reduced pressure furnished the crude product, which was loaded on the silica column and illute with Ethyl acetate-hexane (3 : 7) solvent system to get pure diazepine.

**Representative Spectral Data for 2, 2,4-Trimethyl-2,3-dihydro-1*H*-1,5-benzodiazepine.**

**IR (KBr)**

3290 (NH), 1642 (C=N), 1592 (Ar) cm^−1^;

**^1^H NMR (300 MHz, CDC_l3_)**

*δ* 1.34 (s, 6H, 2C_H3_), 2.20 (s, 2H, C_H2_), 2.36 (s, 3H, C_H3_), 3.45 (brs, 1H, NH), 6.62–7.21 (m, 4H, Ar);

**^13^C NMR (300 MHz, CDCl_3_)**

*δ* 171.6, 140.4, 138.0, 126.9, 125.3, 121.8, 121.6, 68.1, 45.4, 30.6, 29.5, 29.5;

**GC/MS**

M^+^ 188. Mp 125 °C.

## Figures and Tables

**Table 1:** Influence of catalysts on preparation of 1,5-benzodiazepines from o-phenylendiamine and acetone^a^.

Entry	Catalyst	Time	Yield^b^
1	—	20 h	trace
2	HCl	3 h	79
3	SiO_2_	3 h	91
4	YbCl_3_	1.5 h	85
5	[HMIm]BF_4_	3 h	43
6	Amberlyst-15	3.5 h	90
7	Polyphosphoric acid	3 h	83
8	Bismuth (III) salts	1 h	95

^a^Reaction condition: *o*-phenylendiamine (0.01 mol), acetone (0.02 mol), Bismuth (III) salts (0.002 mol), Ionic liquid (2 mL), and room temperature. ^b^Isolated Yield.

**Table 2:** Bismuth (III) salts catalysed condensation of *o*-phenylendiamine with different ketones^a^.

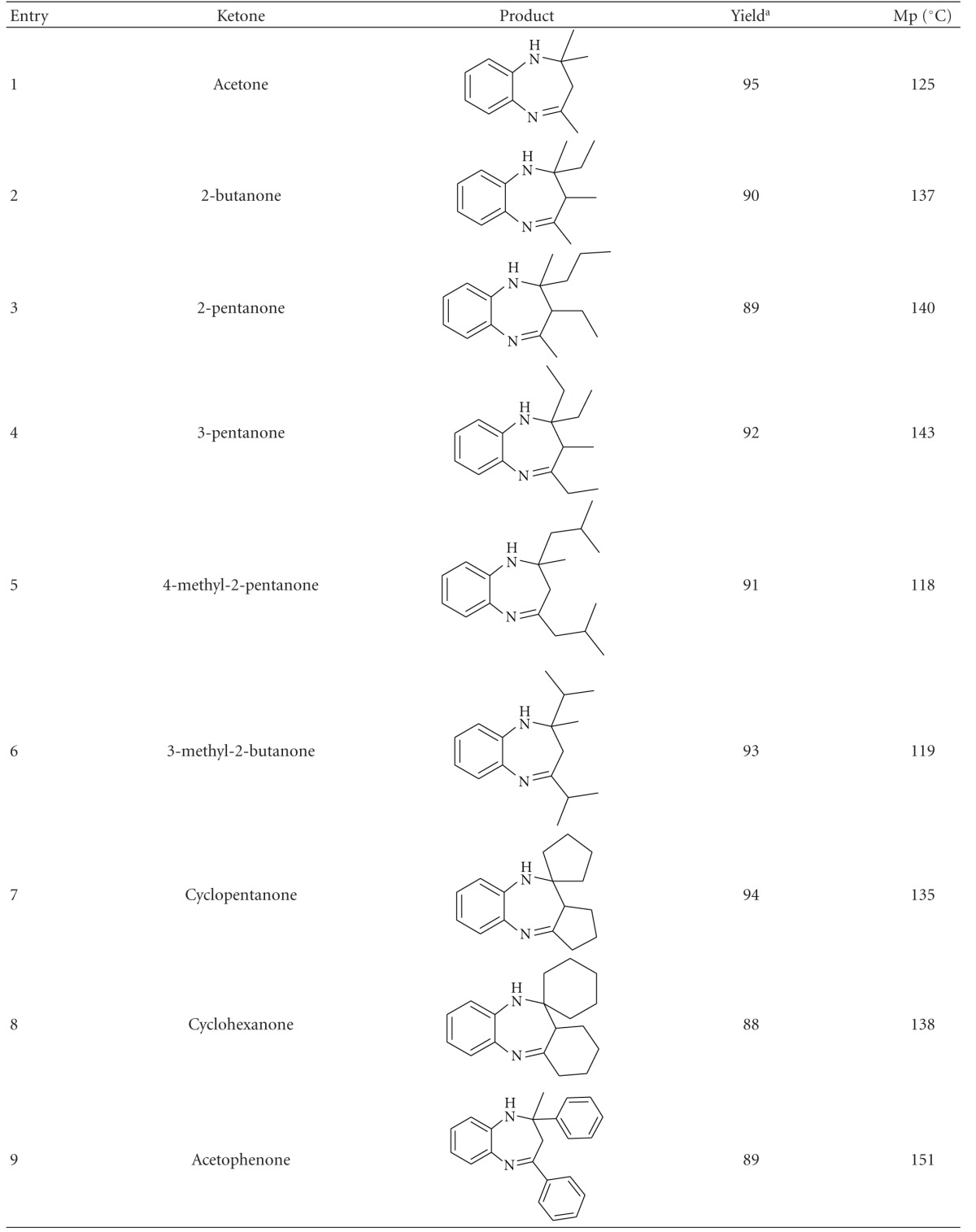

^a^Reaction conditions: *o*-phenylenediamine (0.01 mol), ketones (0.02 mol), Bismuth (III) salts (0.002 mol), ionic liquid (2 mL), room temperature, and time 1 hr. ^b^Isolated Yield.
